# Shedding light on the function of autophagy in complicated pregnancies

**DOI:** 10.1038/s41419-025-08311-7

**Published:** 2026-01-08

**Authors:** Shuting Wan, Yuxing Huang, Huixia Yang

**Affiliations:** 1 Department of Obstetrics and Gynecology, Peking University First Hospital, Beijing, China; 2 Beijing Key Laboratory of Maternal Fetal Medicine of Gestational Diabetes Mellitus, Beijing, China

**Keywords:** Nutrient signalling, Cell death and immune response, Gestational diabetes, Predictive markers, Macroautophagy

## Abstract

Autophagy is a conserved degradation process in eukaryotic cells that is regulated by autophagy-related genes. During autophagy, lysosomes break down cytoplasmic proteins and damaged organelles. This process plays a pivotal role in cell growth and development, protection against metabolic stress and oxidative damage, and the maintenance of cellular homeostasis through the recycling of cellular components. Pregnancy encompasses crucial events such as decidualization, embryo implantation, and fetal growth. Abnormal autophagy has been implicated in several pregnancy complications and can significantly impact both maternal and fetal health. Understanding the relationship between autophagy and complicated pregnancies could open new avenues for potential therapeutic interventions to improve maternal and fetal outcomes. In this review, we summarize the intricate relationship between autophagy and pregnancy complications, elucidate the role of autophagy in gestation, and discuss the clinical significance of autophagy in mitigating or preventing pregnancy-related disorders.

## Facts

- Autophagy, as a conserved lysosome-mediated degradation mechanism, is being intensively investigated by researchers to elucidate its dual role in disease pathogenesis.
- Dysregulated autophagy leads to immune dysregulation, vascular injury, and apoptosis.
- Recurrent implantation failure, preeclampsia, gestational diabetes mellitus, fetal growth restriction, and recurrent spontaneous abortion are common pregnancy complications that can lead to severe adverse pregnancy outcomes, imposing significant burdens on affected individuals and their families.
- Accumulating evidence has demonstrated that autophagy is involved in the pathogenesis and progression of several pregnancy-related disorders.

## Open questions

- What pivotal functions does autophagy serve in preserving homeostatic balance at the maternal–fetal interface?
- What are the underlying mechanisms through which autophagy contributes to the pathogenesis of various pregnancy complications?
- In what ways can targeting autophagy pathways mitigate adverse pregnancy outcomes?
- What are the potential approaches and challenges associated with modulating autophagy as a therapeutic strategy for pregnancy complications?

## Introduction

**Fig. 1:**
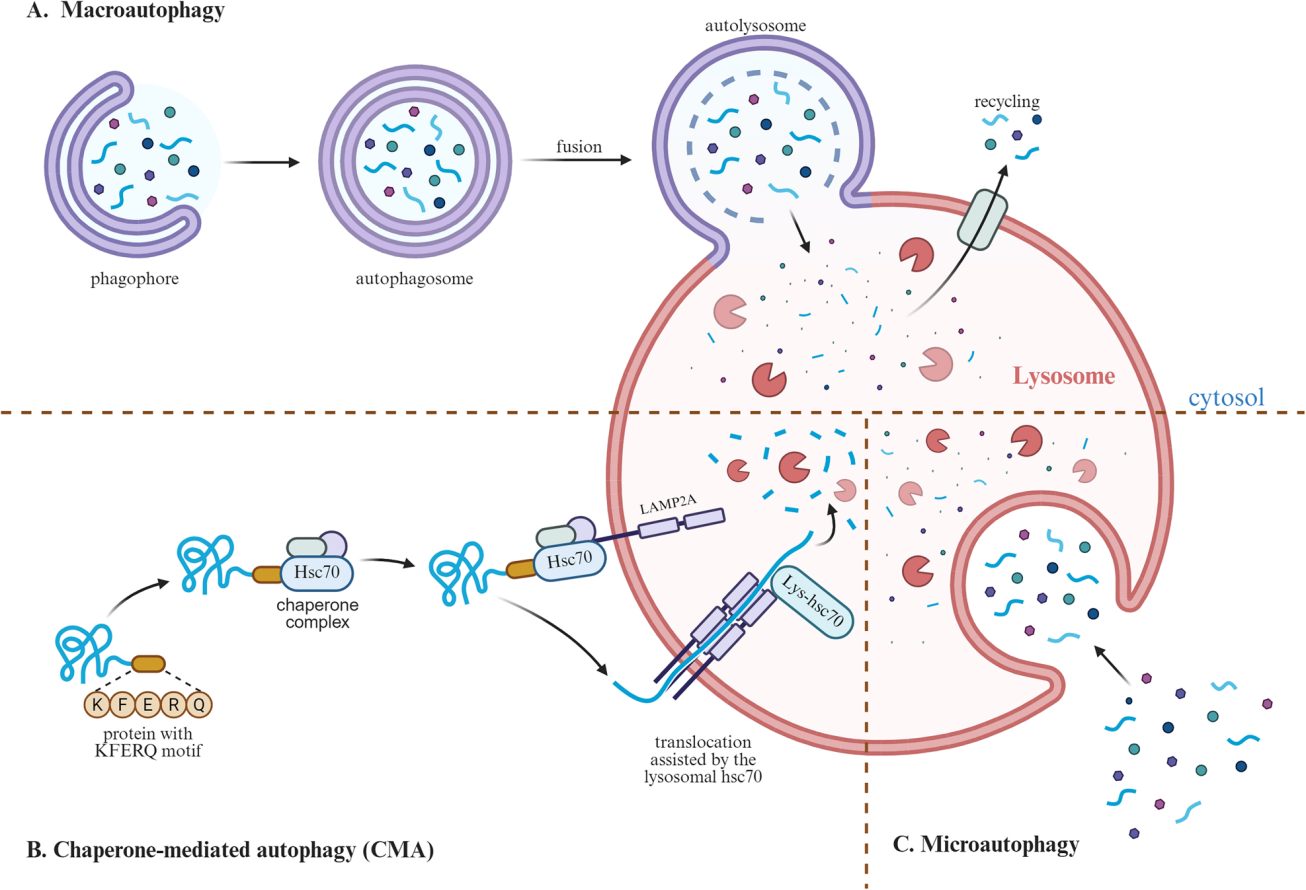
Types of autophagy. **A** Macroautophagy: under the stimulation of external signals, the phagophore forms and encloses the cargo to be degraded and then seals to form an autophagosome. The autophagosome fuses with a lysosome to form an autolysosome, where the contents are degraded and the breakdown products are recycled and reused by the cell. **B** Chaperone-mediated autophagy (CMA), in which the cytosolic chaperone Hsc70 recognizes and binds to the specific KFERQ-like motif of the target protein. The resulting complex is then transported to the lysosomal membrane, where it binds to the membrane receptor LAMP-2A. Subsequently, LAMP-2A oligomerizes to form a translocation channel. With the assistance of chaperones, the target protein unfolds and traverses the channel into the lysosomal lumen, where it is ultimately degraded by hydrolytic enzymes, and the breakdown products are recycled by the cell. **C** In microautophagy, lysosomes engulf cytoplasmic components (such as damaged proteins or organelles) through invagination, budding, and deformation of their own membrane. The engulfed material forms vesicles within the lumen, where it is degraded by hydrolytic enzymes, and the resulting products are recycled for cellular use. LAMP-2A, Lysosome-Associated Membrane Protein 2A.

Autophagy is an evolutionarily conserved process that enables the degradation and recycling of cellular components, including macromolecules and damaged organelles [1]. Recently, autophagy has garnered significant attention because of its involvement in the pathology of various diseases [2–4]. For example, defects in autophagy have been implicated in neurodegenerative disorders [5], metabolic diseases [6], infectious diseases [7], and tumors [8]. Macroautophagy, one of the main types of autophagy, involves the formation of double-layered membrane structures called “autophagosomes” [9, 10] (Fig. 1A). These autophagosomes encapsulate cytosolic components such as protein aggregates and damaged or senescent organelles. Eventually, the autophagosomes fuse with lysosomes (called autolysosomes), leading to the degradation and recycling of the enclosed contents [11]. Autophagy is a type of chaperone-mediated autophagy (CMA) that directly degrades target proteins mediated by the Lys-Phe-Glu-Arg-Gln (KFERQ) motif with the assistance of the chaperone heat shock cognate 70 (Hsc70). The receptor protein lysosome-associated membrane protein 2A (LAMP2A) on the lysosomal membrane recognizes the exposed KFERQ motif on the binding protein, guiding the target protein into the lysosome for degradation [11] (Fig. 1B). On the other hand, microautophagy occurs through the direct invagination of lysosomes into vesicles. These vesicles encapsulate a portion of the cytoplasm and deliver it to lysosomes for degradation. Unlike macroautophagy, microautophagy does not involve the formation of independent bilayer membrane autophagosomes [12] (Fig. 1C).

Pregnancy is a complex physiological process that involves dynamic interactions between the mother and the fetus [13]. Certain pregnancies may be accompanied by severe complications, such as preeclampsia (PE) [14], gestational diabetes mellitus (GDM) [15], fetal growth restriction (FGR) [16], and recurrent spontaneous abortion (RSA) [17], which pose significant threats to maternal and fetal health. PE is characterized by the onset of new hypertension and proteinuria after 20 weeks of gestation. The pathological mechanisms of PE involve placental hypoperfusion and endothelial dysfunction [18]. PE may lead to maternal liver and kidney damage [19], HELLP syndrome [20], and eclampsia while increasing the risk of fetal preterm birth, FGR, and perinatal mortality [21]. GDM refers to glucose metabolism abnormalities that are first diagnosed during pregnancy and are closely associated with insulin resistance and pancreatic β-cell dysfunction [15]. It may increase the risk of maternal gestational hypertension and PE [22] but also increase the risk of fetal macrosomia, type 2 diabetes [23], obesity, neonatal hypoglycemia [24], and long-term metabolic syndrome [25]. FGR is defined as a condition in which the fetus fails to reach its growth potential because of pathological factors. FGR is commonly associated with placental insufficiency, which can be caused by maternal hypertension, malnutrition, or placental abruption. This condition increases the risk of fetal perinatal death, neurodevelopmental delays, and metabolic syndrome [26]. Miscarriage refers to the spontaneous termination of pregnancy before 20 weeks, with common causes including chromosomal abnormalities [27], maternal endocrine disorders [28], infections [29], and uterine anatomical anomalies [30]. Recurrent spontaneous abortion (RSA) is a specific subtype of miscarriage that causes significantly greater psychological and physical harm to women than sporadic miscarriage does [31]. Management strategies for these complications include lifestyle interventions, pharmacological treatments (e.g., insulin and magnesium sulfate), and timely termination of pregnancy. Effectively treating pregnancy complications and improving adverse pregnancy outcomes has become an urgent issue that requires attention.

During pregnancy, autophagy plays a critical role in maintaining the dynamic balance at the maternal–fetal interface. First, autophagy promotes the differentiation and fusion of trophoblast cells, maintains the function of syncytiotrophoblasts, and suppresses excessive apoptosis, thereby supporting placental development [32]. Moreover, autophagy enhances the invasive capacity of trophoblasts, facilitates spiral artery remodeling, and ensures placental perfusion and proper formation [33]. Furthermore, autophagy critically promotes maternal immune tolerance toward semiallogeneic fetuses by enhancing dNK cell function [34, 35], facilitating Treg/Th2-biased immunity, driving M2 macrophage polarization, and suppressing proinflammatory responses, thereby maintaining a homeostatic microenvironment at the maternal–fetal interface [36]. Additionally, during early placentation, autophagy mitigates oxidative stress (OS) by removing damaged mitochondria via mitophagy, reducing excessive reactive oxygen species (ROS) levels, and preventing trophoblast dysfunction [37, 38]. Moreover, as a central nutrient sensor in the placenta, mTOR coordinates metabolic adaptation via mTORC1, inhibiting autophagy under nutrient sufficiency and activating it during deprivation to degrade intracellular components for energy and sustain cell survival [39, 40]. Through these multifaceted mechanisms, autophagy plays an essential and integrative role in ensuring normal placental formation and function.

While autophagy plays a critical role in regulating the immune microenvironment at the maternal–fetal interface and in placental OS as well as nutrient sensing, its dysregulation can lead to pregnancy complications. Therefore, in-depth research on the mechanisms of autophagy during pregnancy is crucial for understanding the physiological and pathological processes of gestation.

## Autophagy in recurrent implantation failure

**Fig. 2:**
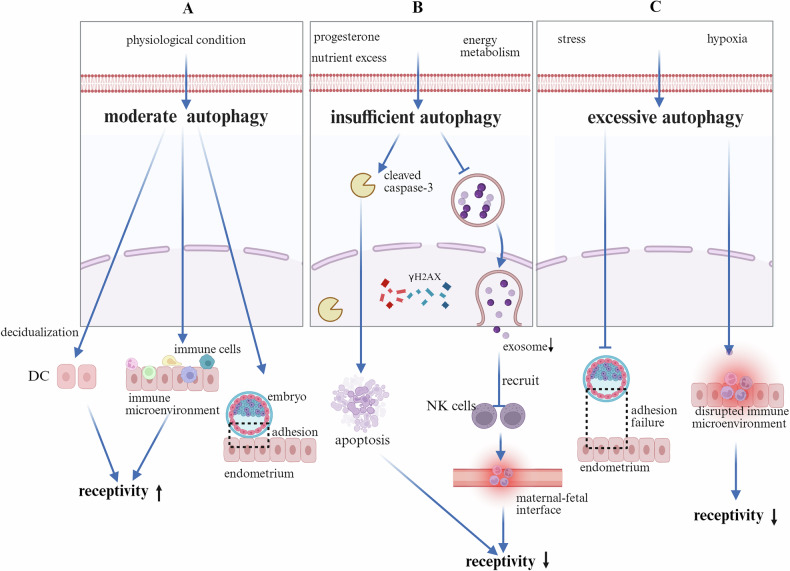
The function of autophagy in the endometrium. **A** Moderate autophagy promotes the decidualization of endometrial stromal cells, contributes to the maintenance of immune homeostasis at the maternal–fetal interface, and thereby improves endometrial receptivity, thus facilitating successful embryo implantation. **B** Hypoxia or ROS induces excessive autophagy, which compromises endometrial decidualization. Excessive autophagy promotes an inflammatory response at the maternal–fetal interface, which impairs endometrial receptivity and results in embryo implantation failure. **C** Impaired autophagy induced by mTOR pathway activation under conditions of hypoxia or ROS leads to a reduction in exosome secretion from ECs. A decrease in autophagy attenuates the recruitment of NK cells, thereby disrupting the immunological homeostasis of the endometrial microenvironment. Moreover, the suppression of autophagy hampers ESC decidualization and ultimately triggers apoptotic pathways, resulting in increased cell death and impaired embryo implantation. ROS Reactive Oxygen Species, EC Endometrial Cell, ESC Endometrial Stromal Cell.

A fertilized egg, or zygote, is formed through the fusion of a spermatozoon and an oocyte, initiating mitotic divisions that lead to blastocyst formation [41]. The blastocyst is then implanted into the receptive endometrium, a process regulated by molecular interactions and endometrial decidualization, which are critical for successful pregnancy establishment [42]. Successful embryo implantation requires a synchronous and coordinated interaction between the embryo and the endometrium [43]. Structural and chromosomal abnormalities, as well as impaired endometrial receptivity, contribute to unexplained RIF [44, 45]. However, the systematic characteristics of endometrial dysfunction during the window of implantation in patients with unexplained RIF remain incompletely understood. Although no consensus has been reached regarding changes in the role of autophagy in RIF, evidence has indicated that autophagy plays a crucial role in embryo implantation by maintaining endometrial cellular homeostasis, regulating the immune microenvironment, and participating in the decidualization process (Fig. 2A).

Disruptions in progesterone signaling, in conjunction with nutrient excess and dysregulated energy metabolism [46, 47], may reduce the LC3-II/I ratio; downregulate the expression of key autophagy-related proteins such as Beclin-1, ATG5, and ATG7; and lead to the accumulation of the autophagy substrate p62. This autophagy dysregulation contributes to RIF. Dysregulated autophagy may impair exosome biogenesis and secretion—key processes in intercellular communication—thereby attenuating the recruitment of immunomodulatory CD49a^+^CXCR4^+^ natural killer (NK) cells. The depletion of these regulatory NK cells disrupts immunological homeostasis within the endometrium, ultimately impairing endometrial receptivity and successful embryo implantation [48, 49]. Taken together, these findings suggest that autophagy regulates RIF by maintaining endometrial cellular homeostasis and modulating the immune microenvironment (Fig. 2B).

OS or hypoxia dysregulates autophagy-related pathways in endometrial cells. This dysregulation leads to an abnormal increase in autophagic flux, which ultimately impairs embryo adhesion and results in implantation failure [50–52]. Furthermore, excessive autophagy disrupts the maternal–fetal immune microenvironment and contributes to impaired endometrial receptivity and unsuccessful embryo implantation [53](Fig. 2C).

These findings highlight the dual role of autophagy—adaptive under physiological conditions but detrimental when dysregulated—which may serve as a therapeutic target for improving implantation outcomes.

## Autophagy in preeclampsia

**Fig. 3:**
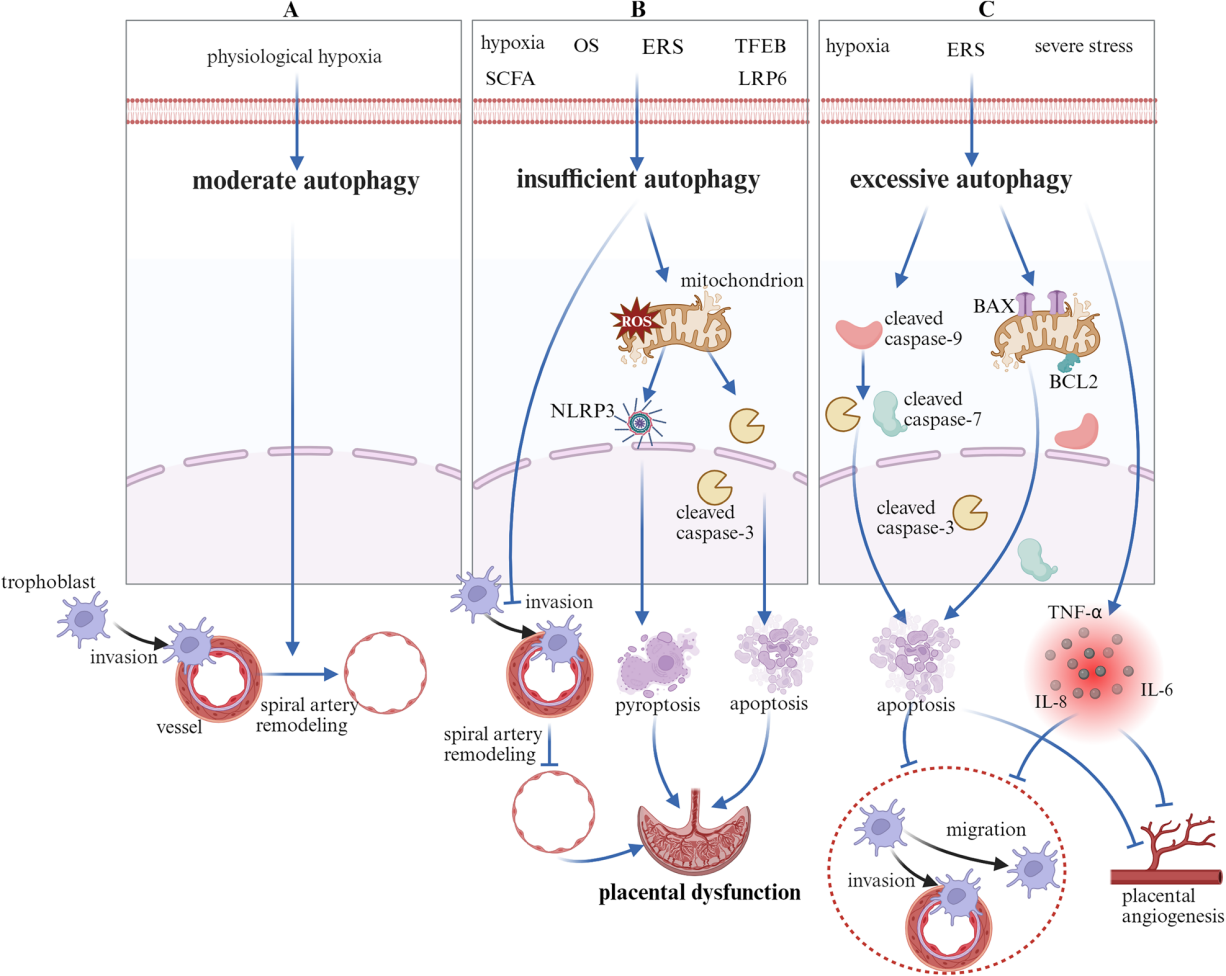
The involvement of autophagy in the pathogenesis of PE. **A** Under pathological conditions, moderate autophagy enhances the invasiveness of trophoblast cells and facilitates the remodeling of the spiral artery. **B** Abnormal changes in hypoxia, OS, ERS, SCFAs, TFEB, and LRP6 trigger autophagy deficiency. On the one hand, this directly impairs trophoblast cell invasion and inhibits spiral artery remodeling. On the other hand, it leads to mitochondrial damage and the accumulation of ROS, both of which collectively activate pyroptosis and apoptosis in trophoblast cells, resulting in placental dysfunction. **C** In response to hypoxia, severe stress, and ERS, excessive autophagy occurs in cells. This activates caspase-3/7/9 and increases the ratio of the pro-apoptotic protein BAX to the anti-apoptotic protein BCL2, leading to excessive apoptosis in trophoblast cells and inhibition of their invasive and migratory capabilities. Simultaneously, excessive autophagy directly triggers the release of large amounts of inflammatory factors, suppressing placental angiogenesis. OS Oxidative Stress, ERS Endoplasmic Reticulum Stress, SCFAs Short-Chain Fatty Acids, TFEB Transcription Factor EB, LRP6 LDL Receptor Related Protein 6, ROS Reactive Oxygen Species, BAX BCL2-associated X protein, BCL2 B-cell lymphoma 2.

Preeclampsia (PE), a multisystem pregnancy disorder, is defined as new-onset hypertension (SBP ≥ 140 mmHg and/or DBP ≥ 90 mmHg) after 20 weeks of gestation accompanied by proteinuria, end-organ dysfunction, or both [54, 55]. PE can be classified into two subtypes on the basis of the time of onset, namely, early onset (delivery before 34 weeks) and late onset (delivery after 34 weeks) []. Early-onset PE is primarily associated with abnormal placental development, including impaired spiral artery remodeling and reduced placental perfusion. It is often accompanied by FGR and tends to have a more prolonged clinical course. Late-onset PE is linked to excessive placental growth, compression of the intervillous space, and subsequent hypoxia. Late-onset PE is considered a manifestation of placental aging in late pregnancy, is more common clinically, and is often associated with unexplained stillbirth or late-onset FGR. Both types present with maternal hypertension and proteinuria, along with similar biomarker changes, such as elevated sFlt-1 and reduced PlGF levels [56]. Moreover, both early-onset and late-onset PE ultimately manifest as stress responses in the STB, including OS, ERS, mitochondrial dysfunction, apoptosis, and autophagy, which constitute the core mechanism underlying the disease [57]. Autophagy plays a pivotal role in PE. For example, appropriate upregulation of autophagy is observed in first-trimester placentas under physiological hypoxia, where it supports trophoblast invasion and vascular remodeling while protecting trophoblasts from cell death induced by hypoxia or nutrient deficiency [58](Fig. 3A). However, whether insufficient autophagy or excessive autophagy contributes to both early-onset and late-onset PE remains unclear.

Autophagy suppression is synergistically driven by multiple upstream factors. First, hypoxia/OS impairs mitochondrial function and activates the mTOR pathway through ROS accumulation [59]. Second, transthyretin aggregation induces excessive unfolded protein response activation, causing ERS that disrupts autophagic flux–lysosomal coordination [60]. Third, metabolic disturbances characterized by deficient short-chain fatty acids [61] and dysregulation of key autophagy regulators such as TFEB and LRP6 further exacerbate autophagic defects [62, 63]. The resulting autophagy dysfunction impaired trophoblast invasion and vascular remodeling capabilities alongside failed spiral artery transformation [59]. Critically, inhibition of autophagy triggers a self-amplifying cell death cascade in placental trophoblasts, characterized by the activation of caspase-3-dependent apoptosis and NLRP3-mediated pyroptosis secondary to mitochondrial dysfunction and ROS accumulation. This synergistic induction of multiple cell death pathways collectively accelerates placental dysfunction and compromises trophoblast homeostasis [60, 64, 65] (Fig. 3B). In addition to the relationship between autophagy and cell death pathways, autophagy promotes ferroptosis through ferritinophagy, mitophagy, and lipophagy. However, autophagy also attenuates ferroptosis by clearing ROS and damaged mitochondria [66]. Moreover, ferroptosis feedback regulates autophagic activity via OS and the Nrf2 pathway [67, 68]. These two processes act synergistically in various physiological and pathological contexts, not only serving as cellular stress defense mechanisms but also potentially cooperating to promote cell death.

In the pathogenesis of PE, placental tissues consistently exhibit a state of overactivated autophagy [69, 70]. This abnormal enhancement is driven primarily by key upstream inducers, such as hypoxia [70, 71], ERS [70], and OS [72]. These factors disrupt downstream molecular mechanisms, resulting in activation of the cGAS-STING pathway [73], HIF-1α [74], and PI3K/AKT/mTOR signaling [75] and inhibition of SIRT1/AMPKα/mTOR signaling [76]. Among the inducers of PE, hypoxia serves as a central trigger, stabilizing factors such as HIF-1α and coordinating multiple signaling cascades to collectively promote excessive autophagy [74]. Moreover, excessive autophagy is not protective but induces apoptosis through activated caspase-3/7/9, mitochondrial membrane potential collapse, and an elevated BAX/BCL-2 ratio [69, 71]. Moreover, hyperactivated autophagy increased the release of inflammatory cytokines, such as TNF-α, IL-6, and IL-8 [71], thereby impairing trophoblast migration, invasion, and placental angiogenesis [72, 77] (Fig. 3C).

Autophagy plays a dual role in PE. Under physiological conditions, moderate autophagy is essential for placental development and cytoprotection. However, under pathological conditions, its dysregulation contributes to the pathogenesis of PE by disrupting trophoblast function, inducing various types of cell death, and triggering inflammatory responses. Owing to the difficulty in clarifying the specific mechanisms of autophagy dysregulation in PE and the lack of in vivo dynamic monitoring methods, determining whether autophagy is a causative factor or merely a secondary phenomenon remains challenging.

## Autophagy in gestational diabetes mellitus

**Fig. 4:**
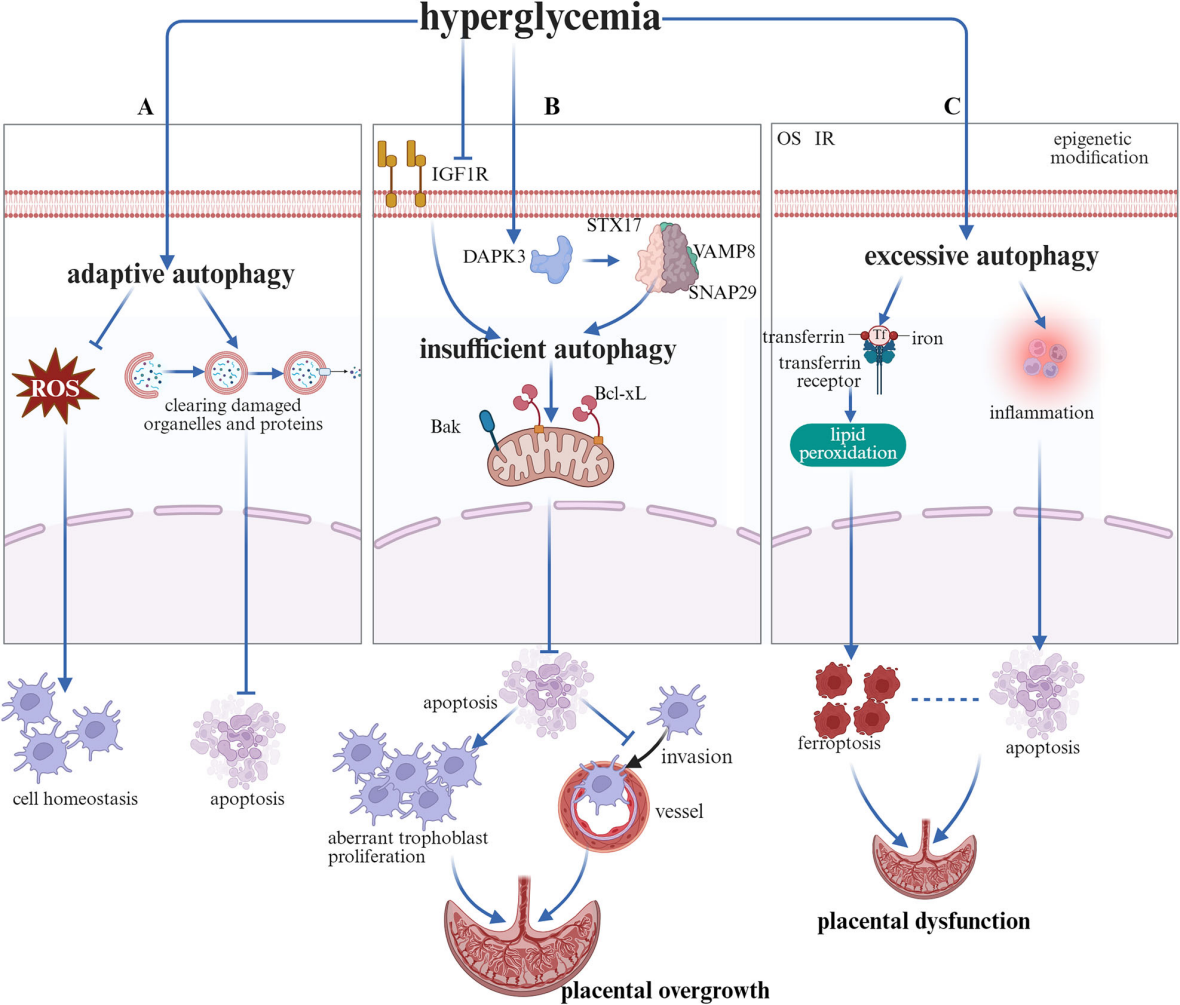
The function of autophagy in GDM. **A** Hyperglycemia triggers adaptive upregulation of placental autophagy, which serves to mitigate the accumulation of intracellular ROS induced by elevated glucose levels and helps maintain cellular homeostasis. Furthermore, enhanced autophagic activity facilitates the removal of damaged organelles and misfolded proteins, thereby preventing excessive trophoblast apoptosis. **B** Hyperglycemia induces intracellular OS, placental IR and abnormal epigenetic modifications, all of which collectively contribute to the activation of excessive autophagy. This pathological increase in autophagy leads to intracellular iron accumulation, promoting lipid peroxidation and ultimately resulting in trophoblast ferroptosis. Concurrently, it exacerbates inflammatory responses and stimulates trophoblast apoptosis. Although the precise molecular mechanisms underlying the crosstalk between ferroptosis and apoptosis remain to be fully elucidated, these two forms of regulated cell death synergistically contribute to placental dysfunction. **C** Hyperglycemia suppresses IGF1R expression levels but increases intracellular DAPK3 levels, promoting abnormal upregulation of the STX17–VAMP8–SNAP29 complex. Both factors collectively inhibit autophagic activity. Autophagy inhibition increases the ratio of the antiapoptotic protein Bcl-xL to the proapoptotic protein Bak, suppresses trophoblast apoptosis, leads to abnormal proliferation of trophoblasts, and simultaneously inhibits their invasive ability, resulting in placental overgrowth. ROS Reactive Oxygen Species, IR Insulin Resistance, IGF1R Insulin-like Growth Factor 1 Receptor, DAPK3 Death-Associated Protein Kinase 3, STX17 Syntaxin 17, VAMP8 Vesicle-associated membrane protein 8, SNAP29 Synaptosome-associated protein 29, Bcl-xL B-cell lymphoma-extra large, Bak BCL2-antagonist/killer.

GDM, a common complication of pregnancy, is defined as hyperglycemia that is first discovered during gestation and poses risks to maternal and fetal health worldwide [78]. Like other pregnancy complications, autophagy is involved in the pathogenesis of GDM. It primarily functions by inhibiting intracellular ROS accumulation and maintaining cellular homeostasis while degrading damaged proteins and organelles to suppress apoptosis (Fig. 4A).

Hyperglycemia suppresses autophagy through a dual mechanism. On the one hand, it downregulates the insulin-like growth factor 1 receptor (IGF1R), which inhibits ATG7-mediated initiation of autophagy and simultaneously upregulates death-associated protein kinase 3 (DAPK3). DAPK3 promotes the interaction between Synaptosome-associated protein 29 (SNAP29) and Syntaxin 17 (STX17), leading to the formation of an aberrant STX17–SNAP29–Vesicle-associated membrane protein 8 (VAMP8) complex. This pathological complex interferes with the proper fusion of autophagosomes and lysosomes, thereby impairing autophagic flux [79]. Consequently, autophagic activity is impaired, as indicated by a reduced LC3-II/I ratio, decreased Beclin-1 and ATG5 expression, and the accumulation of the autophagy substrate p62 [80]. On the other hand, autophagy suppression elicits pathological effects in a tissue-specific manner. In murine pancreatic β-cells, it leads to increased apoptosis and reduced insulin secretion, thereby aggravating insulin resistance and metabolic dysfunction [81]. In the placenta, compromised autophagy decreases apoptosis through upregulation of the antiapoptotic protein Bcl-xL and downregulation of the proapoptotic factor Bak [80]. This dysregulation promotes aberrant trophoblast proliferation, impairs invasion, and results in placental overgrowth, ultimately contributing to fetal macrosomia or large-for-gestational-age neonates (Fig. 4B).

Hyperglycemic conditions in the context of GDM significantly induce excessive autophagy through multiple mechanisms, including downregulation of the GATA2/FGF21 axis [82], activation of the circCDH2/miR-33b-3p/ULK1 regulatory axis [83], suppression of the miR-193b/IGFBP5 axis [84], and activation of the SIRT3/AMPK/mTOR pathway [85]. The primary upstream triggers stem from hyperglycemia-induced OS, which leads to a significant accumulation of ROS and is further exacerbated by concomitant insulin resistance. Moreover, these effects are also modulated by epigenetic modifications. For example, epigenetic regulation through 5hmC modifications is enriched in the promoter regions of key genes (such as ATG5), modulating autophagy and apoptosis levels in trophoblasts, thereby affecting OS, ROS accumulation, and insulin signaling pathways. [86] (Fig. 4C).

The downstream pathological consequences of GDM play a complex dual role. On the one hand, initial moderate autophagy enhancement may serve as an adaptive response, mitigating OS pressure and suppressing apoptosis by clearing damaged organelles. For instance, this can occur by reducing the expression of the proapoptotic proteins Bax and cleaved caspase-3 and upregulating the antiapoptotic protein Bcl-2 [82]. On the other hand, under specific signaling pathways, such as the SIRT3/AMPK/mTOR-mediated cascade, excessive activation of autophagy can further induce iron accumulation via transferrin receptors and transferrin, lipid peroxidation, and depletion of glutathione, ultimately leading to the induction of ferroptosis [85]. Furthermore, this process may interact with inflammation-associated pyroptosis through mechanisms that remain to be elucidated, thereby synergistically contributing to placental dysfunction and adverse pregnancy outcomes.

## Autophagy in fetal growth restriction

FGR is defined as a fetus failing to reach its growth potential because of pathological factors and can be categorized as maternal, fetal, or placental [87]. It is a common complication during pregnancy and is associated with various adverse perinatal outcomes. Estimating fetal growth potential can be challenging, and continuous assessments to monitor a decrease in the fetal weight percentile can be difficult [88]. As a result, doctors often provide a “snapshot” of estimated fetal weight (EFW) at specific points in time.

**Fig. 5:**
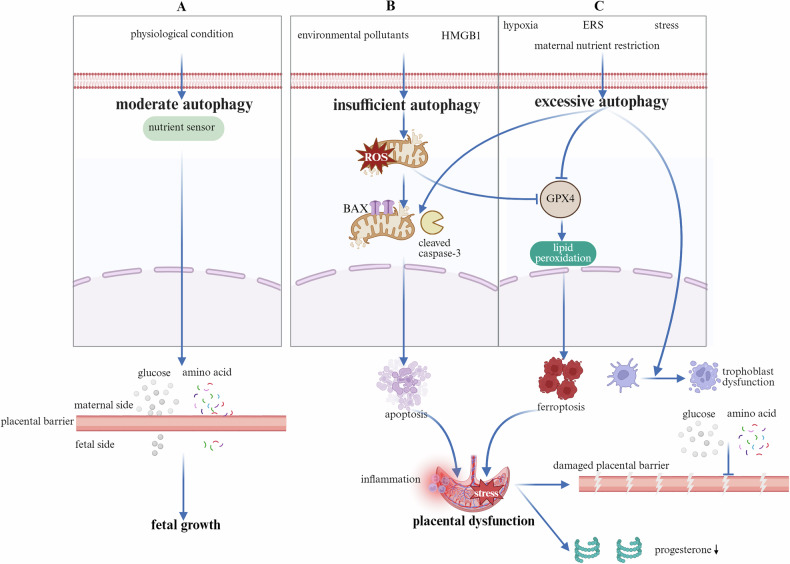
The function of autophagy in FGR. **A** Under physiological conditions, moderate autophagy acts as a nutrient sensor within the body, facilitating the transport of maternal nutrients such as glucose and amino acids to the fetus and thereby promoting fetal growth. **B** Upon exposure to environmental pollutants and stimulation by inflammatory factors, autophagy is inhibited. This leads to mitochondrial damage and the accumulation of ROS within cells. On the one hand, this activates the caspase-3 and BAX-dependent apoptotic pathways, promoting placental apoptosis. On the other hand, it suppresses the expression of intracellular GPX4, promoting lipid peroxidation and leading to ferroptosis. Both ferroptosis and apoptosis collectively contribute to placental dysfunction, disrupt the placental barrier, and impair nutrient transport. Moreover, placental dysfunction results in reduced progesterone secretion. **C** Excessive autophagy occurs under conditions such as hypoxia, ERS, OS, and maternal nutrient restriction. This directly causes trophoblast dysfunction and stimulates the same apoptotic and ferroptosis pathways, resulting in placental dysfunction, disruption of the placental barrier, and impairment of nutrient transport and progesterone synthesis. ROS Reactive Oxygen Species, BAX BCL2-associated X protein, GPX4 Glutathione Peroxidase 4, ERS Endoplasmic Reticulum Stress, OS Oxidative Stress.

Small for gestational age (SGA), defined as an estimated fetal weight or abdominal circumference less than the 10th or 3rd percentile for gestational age, is typically used to clinically evaluate FGR [26, 89]. FGR not only results in decreased fetal weight at birth but has also been linked to metabolic syndromes in adulthood, such as obesity, diabetes, cardiovascular illness, and hypertension [90]. Despite differences in pathophysiologic mechanisms, the core issues associated with FGR are impaired uterine–placental perfusion and insufficient fetal nutrition. Autophagy serves as a crucial cellular mechanism for detecting and responding to nutrient deprivation. Given that the placenta functions as a vital organ for nutrient exchange between the mother and fetus [91], placental autophagy is intricately involved in the pathophysiology of FGR (Fig. 5A).

Alterations in autophagy levels in FGR remain controversial, and no unified conclusion has yet been reached. At one extreme, placental autophagy is generally suppressed, characterized by a reduced LC3-II/I ratio and P62 accumulation [92]. Key etiological factors include exposure to environmental pollutants, such as PM2.5, which inhibits AMPK/mTOR activity [93]; bisphenol A (BPA), which induces OS and ERS [94]; carbon black nanoparticles, which disrupt mitochondrial dynamics [95]; and placenta-specific HMGB1 knockout, which suppresses autophagy via the HMGB1–ERK pathway [92]. This suppression of autophagy leads to the accumulation of intracellular stress, including mitochondrial damage and increased oxidative products, inducing both apoptosis [94], manifested by upregulated caspase-3 and Bax expression, and ferroptosis, characterized by decreased glutathione peroxidase 4 (GPX4) expression and elevated lipid peroxidation [95]. These processes interact synergistically to create an inflammatory microenvironment, which ultimately leads to placental dysfunction. This dysfunction is characterized by impaired nutrient transport, including reduced uptake and transfer of glucose and amino acids, decreased synthesis of essential hormones such as progesterone, and disruption of placental barrier integrity (Fig. 5B).

At the other end of the spectrum, excessive autophagy activation may paradoxically compromise its protective effects and promote the occurrence of apoptosis or ferroptosis [96]. Exposure to environmental pollutants [97, 98], maternal nutrient restriction [99], hypoxia, OS [100], and ERS [101, 102] increase placental autophagy levels in FGR through the suppression of the mTOR signaling pathway [96] or the activation of specific stress-responsive pathways, including the PERK/ATF4 pathway [98], the GCN2/ULK1/FUNDC1 pathway [103], the ERK pathway, and the IRE1α/XBP1 pathway [102]. Enhanced autophagic flux is accompanied by increased degradation of autophagic substrates, particularly mitochondrial components linked to mitophagy, as demonstrated by reduced expression levels of mitochondrial markers, including HSP60, COX IV, and TOM20 [98]. Moreover, excessive autophagy directly impairs trophoblast function by inhibiting proliferation, migration, invasion, and syncytialization [99, 104]. Autophagy further compromises placental barrier integrity and disrupts the transport of essential nutrients such as glucose and amino acids while also suppressing the synthesis of key hormones, notably progesterone [103]. In parallel, excessive autophagy promotes apoptosis through caspase-3 activation and Bax upregulation while also inducing ferroptosis by degrading ferritin, inhibiting GPX4, and enhancing lipid peroxidation. [97]. These concurrent death pathways synergistically exacerbate placental OS and inflammatory microenvironment formation, ultimately severely compromising placental function and fetal development (Fig. 5C).

Autophagy plays a dichotomous role in the pathogenesis of PE and FGR. In PE, diminished autophagic activity compromises vascular function and spiral artery remodeling while concurrently exacerbating inflammatory responses. In contrast, excessive autophagy in FGR contributes to trophoblast dysfunction, increased mitophagy and ferroptosis, and impaired nutrient transport across the placenta. Notably, both conditions are initiated by overlapping upstream stressors, including hypoxia, OS, ERS, and inflammation. These stress signals, however, modulate autophagy in opposing directions. For instance, hypoxia suppresses autophagy in PE through disruption of TFEB signaling [60, 62, 105]. However, in FGR, it promotes mitophagy via activation of the HIF-1α/BNIP3 pathway [96, 104]. Similarly, OS inhibits autophagy in the context of PE but enhances it in FGR, primarily through differential regulation of the AMPK/mTOR signaling cascade [96, 102, 104]. Collectively, these findings suggest that common pathogenic stimuli elicit divergent autophagic responses, ultimately giving rise to distinct clinical phenotypes in PE and FGR. The dual or even opposing roles exhibited by this same biological process across different disease contexts profoundly illustrate the context-dependent nature and regulatory complexity of autophagy in the pathogenesis of diseases.

## Autophagy in recurrent spontaneous abortion

**Fig. 6:**
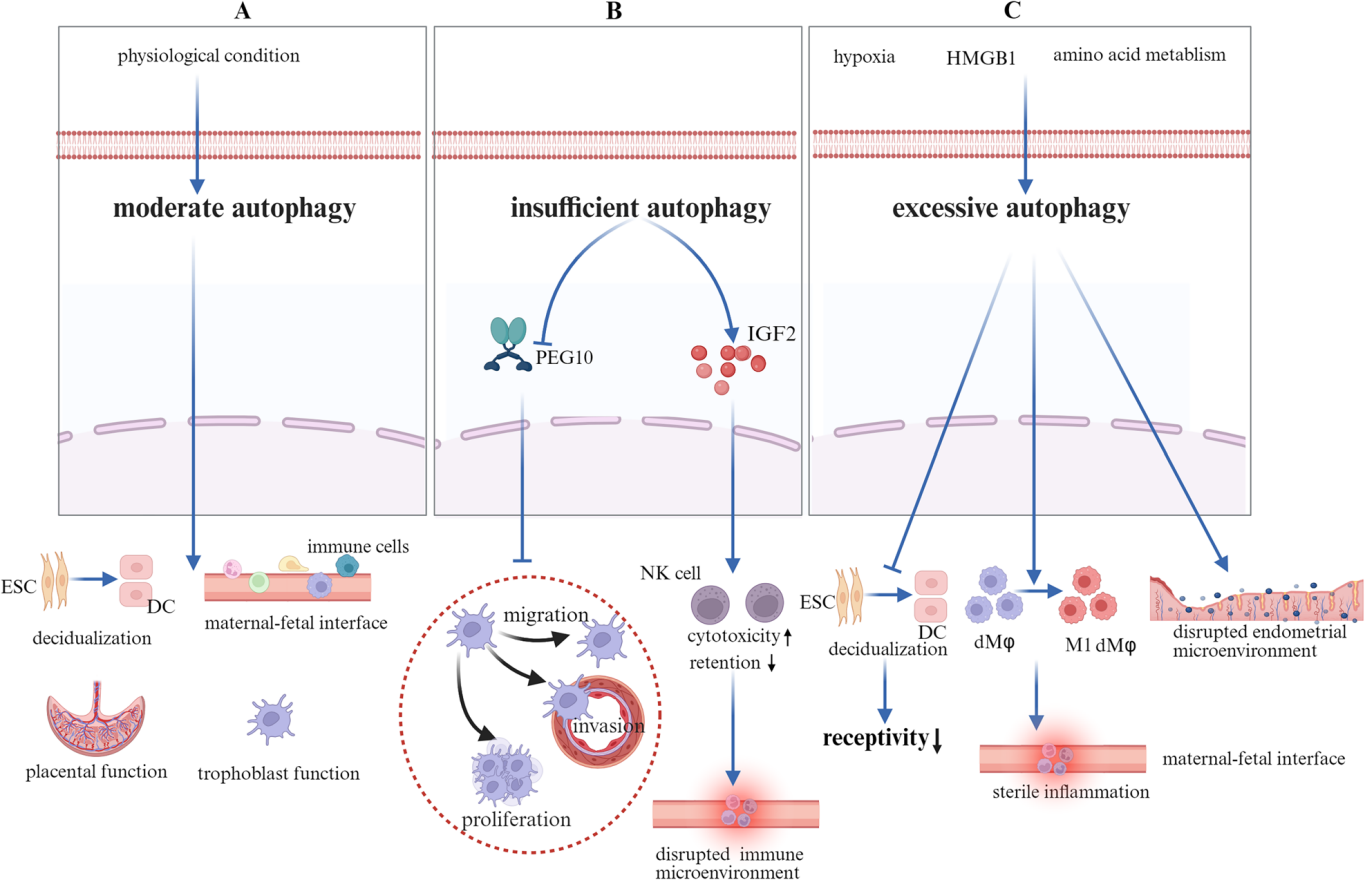
The role of autophagy in RSA. **A** Under physiological conditions, moderate autophagy facilitates the decidualization process, maintains immune balance at the maternal–fetal interface, and supports placental and trophoblast cell function. **B** Autophagy deficiency inhibits the expression of intracellular PEG10 protein, thereby suppressing trophoblast proliferation, invasion, and migration. Moreover, impaired autophagy promotes IGF2 secretion by trophoblasts, leading to enhanced cytotoxicity and reduced residency of decidual NK cells. This disrupts the immune microenvironment at the maternal–fetal interface. **C** Under stimulation by hypoxia, the inflammatory factor HMGB1, and abnormal amino acid metabolism, excessive autophagy occurs, disrupting the endometrial microenvironment, inhibiting endometrial decidualization, and impairing endometrial receptivity. Additionally, excessive autophagy promotes the polarization of macrophages toward the M1 phenotype, triggering sterile inflammation at the maternal–fetal interface and ultimately resulting in RSA. PEG10 Paternally Expressed Gene 10, IGF2 Insulin-like Growth Factor 2, HMGB1 High Mobility Group Box 1 protein.

RSA, clinically defined as two or more consecutive pregnancy losses prior to the 20th gestational week, affects approximately 1% to 5% of women of reproductive age [106]. The pathogenesis of RSA involves a complex interplay of multifactorial contributors, including genetic abnormalities, uterine anatomical defects, endocrine disorders, infectious agents, and immunological dysregulation across diverse pathological dimensions [107]. Autophagy plays a crucial role in establishing and maintaining pregnancy, primarily by facilitating trophoblast function, decidualization, placental development, and maternal–fetal immune homeostasis through its appropriate activation [36] (Fig. 6A). Current research highlights two divergent viewpoints regarding the relationship between autophagy and RSA.

Autophagy deficiency disrupts pregnancy maintenance through two principal mechanisms. First, it downregulates PEG10 expression and impairs trophoblast proliferation, migration, and invasion, thereby compromising normal embryo implantation [108]. Second, it disrupts maternal–fetal immune homeostasis by upregulating IGF-2 expression in trophoblasts, enhancing the cytotoxic activity of dNK cells while reducing their retention [109] (Fig. 6B).

The abnormal upregulation of autophagy may also lead to RSA. For example, hypoxia triggers the activation of autophagy by activating the HIF-1α pathway, resulting in impaired endometrial decidualization and reduced receptivity [51]. OS and inflammatory factors (such as HMGB1), by binding to RAGE/TLR receptors or inhibiting the HDAC–TFEB axis, activate the AMPK/GCN2 or autophagy–lysosome pathways [110], thereby impairing trophoblast function. This impairment manifests as reduced proliferative and invasive capacities, cell cycle arrest, and impaired placental angiogenesis (Fig. 6C).

Dysregulated autophagy contributes to pregnancy loss by impairing critical processes such as maternal–fetal immune tolerance, embryo implantation, endometrial decidualization and receptivity. Currently, autophagy investigations rely on animal models and in vitro cultured cells, which may not fully reflect the human physiological context. Moreover, a comprehensive exploration of the overall regulatory network of autophagy in RSA, as well as the development of targeted clinical intervention strategies, remains insufficient.

## Autophagy in intrahepatic cholestasis of pregnancy

Intrahepatic cholestasis of pregnancy (ICP), a pregnancy-specific hepatobiliary condition, is clinically characterized by persistent pruritus in the absence of primary cutaneous disorders and, pathognomonically, marked elevations in maternal serum bile acid concentrations. This disorder primarily manifests in the third trimester of gestation [111] and affects approximately 1.5–4% of pregnancies [112], leading to adverse outcomes for both mothers and fetuses [113]. These outcomes include PE, GDM, meconium-stained amniotic fluid, premature delivery, neonatal respiratory distress syndrome, and intrauterine fetal death [114]. Autophagic flux has been reported to be significantly inhibited, indicating impaired autophagosome formation or degradation [115, 116]. This suppression is driven primarily by two major upstream mechanisms: bile acids downregulate AMPK/ULK1 signaling [115] and upregulate EGFR/p-EGFR in placental tissue, activating the PI3K/AKT/mTOR pathway [116]. Autophagy inhibition leads to decreased mitochondrial membrane potential and reduced ATP generation, thereby exacerbating cellular energy crisis. Moreover, compromised autophagy enhances the susceptibility of trophoblasts to apoptosis under stress conditions, such as nutrient deprivation, as evidenced by increased expression levels of cleaved caspase-3, PARP, and γ-H2A.X. Using animal models, researchers confirmed that cholestasis combined with starvation synergistically aggravates placental apoptosis. Additionally, autophagy inhibition impedes GPX4 degradation, consequently suppressing ferroptosis and interfering with normal cellular turnover. This dual inhibition of both autophagy and ferroptosis likely contributes to cellular dysfunction and enhanced inflammatory responses [116].

However, the placenta exhibits an enhanced autophagic state in patients with severe ICP, as evidenced by significantly increased LC3 and ATG14 expression levels. Furthermore, these findings reveal that excessive autophagic activation is positively correlated with elevated placental expression of GABRP. These results suggest that GABRP may function as an upstream inducer of enhanced autophagy, potentially contributing to placental dysfunction and ultimately leading to adverse pregnancy outcomes [117].

In the investigation of autophagy in ICP, the animal model, as well as methodological and molecular mechanistic heterogeneity, may affect the reliability of conclusions. For example, by using a comprehensive autophagic flux analysis, the authors provide robust evidence supporting the inhibition of autophagic flux [115]. This approach effectively distinguishes autophagy activation from degradation blockade and is highly reproducible. However, some studies have assessed autophagy activation using only single static markers and have not validated autophagic flux dynamics, thereby reducing the reliability of the conclusions. Moreover, molecular mechanistic heterogeneity may also contribute to these discrepancies, as different regulatory pathways can differentially modulate autophagy.

## The dual role of autophagy in pregnancy complications

Given that the literature reports seemingly contradictory findings regarding the role of autophagy in various pregnancy complications, specific explanations for the underlying causes of this dual functionality are often lacking. Here, we reviewed these studies and attempted to systematically analyze disease stage, experimental cell models involved, key signaling pathways, and specific mechanisms of action. Through this analysis, we aim to clarify the precise contexts under which autophagy plays protective or pathological roles, thereby providing a clear explanatory framework for understanding these conflicting observations (Table 1).

**Table 1:** Summary of the dual role of autophagy in pregnancy complications.

Disease	Autophagy function	Disease stage	Upstream signaling pathway	Cell type	Autophagy mechanism	Refs
**RIF**	protective	mild inflammatory state, during decidualization, embryo implantation stage	HIF-1α, mTOR, Atg9A-mediated mitophagy, FIP200, Wnt	THESCs, EPSC	clearing damaged organelles and abnormal protein aggregates; regulating immune responses and suppressing pro-inflammatory immune activation; sustaining normal progesterone signaling	[49, 51, 53, 121, 122]
	pathogenic	mid-secretory, advanced stage of RIF	IFN-γ, CXCL12/CXCR4, NOTCH1	ESCs	impairment of cellular structure and function	[50, 52]
**PE**	protective	the early stage of PE, mild hypoxia, the early stage of OS	NOS1/NO, JAK2/STAT3, AMPK, IRE1α, TFEB, mTOR, GPR41/AKT, EZH2/mTOR	trophoblast cell lines (JAR, JEG-3, HTR-8/SVneo), placental macrophages	clearance of damaged organelles, abnormal protein aggregates, and oxidative stress products, and inhibition of inflammatory responses; alleviation of ERS; promotion of M2 polarization; inhibition of apoptosis; alleviation of senescence	[60, 61, 65, 123, 124]
	pathogenic	advanced stage of PE, severe OS, chronic hypoxia	IRE1α, NLR3, NF-κB, TLR4/MyD88/MAPK, PTEN/AKT, PPP3/XPO1/TFEB, mTOR/TFEB, BNIP3/PINK1/LC3, cGAS/STING	HTR-8/SVneo, JEG-3, primary human trophoblasts	causing inflammatory responses, cell pyroptosis, and lysosomal dysfunction; increasing protein aggregation and activation of ERS	[64, 65, 73, 105, 125–127]
**GDM**	protective	early stage of IR, early stage of GDM, mild high-glucose environment	IGF1R/ATG7	mouse pancreatic β cells, HTR8/SVneo	clearing intracellular damaged organelles and abnormal proteins; reducing OS, ERS, and apoptosis	[81, 128]
	pathogenic	advancement of GDM, severe high-glucose environment	DAPK3/SNAP29/STX17/VAMP8, FGF21/PI3K/AKT/mTOR, circCHD2/miR-33b-3p/ULK1, AMPK/mTOR, miR-193b/IGFBP5	HTR8/SVneo, HUVECs, pTr2	regulating autophagosome-lysosome fusion; decreased proliferation, migration, and invasion of trophoblast; enhanced OS, inducing cell damage and apoptosis; promoting ferroptosis	[79, 82, 84, 85, 129]
**FGR**	protective	early pregnancy, early stage of environmental exposure, mild nutrient restriction	AMPK/mTOR, PARKIN-mediated mitophagy, ERK, PI3K/AKT/HIF-1α, NBR1/LC3B-p21, p62-Nrf2	HTR-8/SVneo, JEG3	clearing damaged mitochondria; alleviating OS and ERS, and inhibiting apoptosis; promoting the migration, invasion, and proliferation of trophoblasts; attenuation of pyroptosis and cellular senescence	[92, 95, 99, 100, 130, 131]
	pathogenic	mid-to-late gestation, excessive and sustained OS, severe nutrient restriction	AMPK/mTOR, ERK, 3-MA/PI3K, YAP/TAZ, p62/Keap1/Nrf2, PERK/ATF4, GCN2/ULK1/FUNDC1, NF-κB, PERK/IRE1/ATF6	OTCs, HTR-8/SVneo, TEV-1, JEG3, human primary placental trophoblast	exacerbating ERS and increasing cell death; induces placental ferroptosis; inhibiting the migration, invasion, and proliferation of trophoblast; exacerbating cellular senescence; impairs trophoblast syncytialization; mitochondrial dysfunction	[94, 95, 97–100, 103, 131]
**RSA**	protective	embryo implantation stage, initiation stage of decidualization, mild OS	LPA-LPAR1/PPARG, IGF2/TP53/CXCR4, ITGA3/ULK1, MITF/TNFRSF14, MTORC1	DSCs, DICs, THP-1, HTR-8/SVneo	upregulating adhesion molecules; promoting M2 polarization of dMφ and trophoblast invasion; maintaining immune tolerance; enhancing the residence of dNK cells;enhancing the invasion, migration, and proliferation of trophoblasts	[109, 132–137]
	pathogenic	embryo implantation stage, chronic OS	IGF2/TP53/CXCR4,YY1/PVT1/mTOR, Shh, TFEB-dependent autophagy‒lysosome	HTR-8/SVneo, NK-92, THP-1, T HESCs, JAR	enhancing local inflammatory response; impairing trophoblast invasion; promoting M1 polarization and triggering inflammatory responses; insufficient endometrial decidualization	[108, 109, 121, 133, 136–138]
**ICP**	protective	low-concentration bile acids, mild stress	AMPK/ULK1	HTR8/SVneo	clearing damaged organelles and toxic protein aggregates, providing nutrients and energy, alleviating ERS, and inhibiting apoptosis	[115]
	pathogenic	high-concentration bile acids, severe stress	EGFR,GABRP	/	exacerbating mitochondrial dysfunction and increasing cell apoptosis	[116, 117]

*FIP200* Focal Adhesion Kinase Family Interacting Protein of 200 kDa, *Wnt* Wingless-related integration site, *NOTCH1* Neurogenic Locus Homolog Protein 1, *NOS1* Nitric Oxide Synthase 1, *IRE1α* Inositol-requiring enzyme 1 alpha, *GPR41* G Protein-Coupled Receptor 41, *EZH2* Enhancer of Zeste Homolog 2, *MyD88* Myeloid Differentiation Primary Response 88, *MAPK* Mitogen-Activated Protein Kinase, *PTEN* Phosphatase and Tensin Homolog, *PPP3* Protein Phosphatase 3, *XPO1* Exportin 1, *BNIP3* BCL2/adenovirus E1B 19-kDa protein-interacting protein 3, *SNAP29* Synaptosome-Associated Protein 29 kDa, *STX17* Syntaxin 17, *VAMP8* Vesicle-Associated Membrane Protein 8, *FGF2*1 Fibroblast Growth Factor 21, *ERK* Extracellular Signal-Regulated Kinase, *NBR1* Neighbor of BRCA1 gene 1, *3-MA* 3-Methyladenine, *YAP* Yes-Associated Protein, *TAZ* Transcriptional Co-activator with PDZ-binding Motif, *PERK* PKR-like ER Kinase, *ATF4* Activating Transcription Factor 4, *GCN2* General Control Nonderepressible 2, *FUNDC1* FUN14 Domain Containing 1, *LPA* Lysophosphatidic Acid, *LPAR1* Lysophosphatidic Acid Receptor 1, *ITGA3* Integrin Subunit Alpha 3, *MITF* Microphthalmia-Associated Transcription Factor, *TNFRSF14* TNF Receptor Superfamily Member 14, *Shh* Sonic Hedgehog, *ESCs* endometrium stromal cells, *EGFR* Epidermal Growth Factor Receptor, *GABRP* Gamma-Aminobutyric acid A receptor subunit pi, T *HESCs* human endometrial stromal cells, *EPSC* Endometrial Progenitor Stem Cells, UPR Unfolded Protein Response, *pTr2* porcine Trophoblastic cell line, *OTCs* Ovine Trophoblast Cells, *P4* Progesterone, *DSCs* Decidual Stromal Cells, *DICs* Decidual Immune Cells.

The results in the table reveal that autophagy functions as a “double-edged sword” in the context of pregnancy complications. Its effects—whether protective or detrimental—are largely dependent on the stage of disease progression and the degree of external stress. In early stages or under mild stress, autophagy is activated to prevent cellular damage and maintain placental homeostasis, thereby exerting a protective effect. However, with disease progression or increasing stress levels, autophagy may become dysregulated—manifesting as either insufficient or excessive activity—which ultimately contributes to pathological exacerbation. Taken together, therapeutic strategies should not be limited to broadly enhancing or inhibiting autophagy but should instead focus on precise modulation—aiming to restore its physiological balance through targeted regulation of specific pathways at appropriate stages of disease development.

## Clinical implications of modulating autophagy

On the basis of the aforementioned evidence, appropriate autophagic activity at the maternal–fetal interface is critical for preventing pregnancy complications. In recent years, numerous researchers have attempted to target autophagic pathways at this interface to improve adverse pregnancy outcomes. To provide clearer insight, we have summarized therapeutic strategies targeting placental autophagy for specific pregnancy complications (Table 2). Taken together, these findings suggest that modulating autophagy provides a new way to treat pregnancy complications.

**Table 2:** Modulation of autophagy in patients with gestational complications.

Complication	Autophagy Modulator	Target	Autophagy	Refs
RIF	NOTCH1	AKT-mTOR	Inhibition	[52]
PE	Trehalose-like disaccharides 3-MA Esomeprazole *Akkermansia muciniphila* Resveratrol 4-PBA	TFEB PI3K SIRT1 AMPK SIRT1 PERK	Inhibition Inhibition Inhibition Activation Activation Inhibition	[61, 70, 76, 139–141]
GDM	FABP4 PLGF TXNIP	PTEN BNIP3-PINK1-Parkin TRX	Activation Activation Activation	[142–144]
FGR	Circ_0081343 CircCUL1 Cordycepin	PI3K-AKT-HIF-1α hsa-miR-30e-3p, ANXA1 GPX4, HSC70, LAMP-2a	Activation Inhibition Inhibition	[130, 145, 146]
RSA	BSHXD CREB5 miR-20b-5p ASP	AMPK ATG5, LC3, SQSTM1/p62 ATG16L1, ATG7 Beclin1	Activation Activation Activation Activation	[132, 137, 147–152]
	LPA Rapamycin XBP1	LPAR1, PPARγ mTOR TRAF6	Activation Activation Activation	

*4-PBA* 4-Phenylbutyric acid, *FABP4* Fatty Acid-Binding Protein 4, *PLGF* Placental Growth Factor, *TXNIP* Thioredoxin-Interacting Protein, *BSHXD* Bushen Huoxue decoction, *CREB5* CAMP Responsive Element Binding Protein 5, *ASP* Angelica sinensis polysaccharide, *XBP1* X-Box Binding Protein 1, *SIRT1* Sirtuin 1, *PINK1* PTEN-induced putative kinase 1, *TRX* Thioredoxin, *ANXA1* Annexin A1, *TRAF6* TNF Receptor-Associated Factor 6.

Given the pivotal role of autophagy in pregnancy complications, the risk of manipulating autophagy during gestation should be well understood. To date, no definitive threshold for autophagy has been established in any pathological context, beyond which—either above or below—its role shifts from protective to detrimental. Therefore, placental autophagy must be tightly regulated to maintain normal placental development and function. On the one hand, insufficient autophagy disrupts trophoblast syncytialization by disrupting cellular homeostasis, protein degradation, energy metabolism, and fusion-differentiation processes [118]. On the other hand, excessive autophagy triggers mitochondrial-dependent apoptotic pathways, directly inducing cell death [119, 120] while concurrently suppressing cell migration and invasion capabilities and impairing placental angiogenesis [120]. Therefore, precise manipulation of autophagy is critical for proper placental development and function.

Although extensive studies have demonstrated the crucial role of autophagy in pregnancy, notably, no clinical trials currently target the modulation of autophagy in pregnancy-related conditions. However, we believe it is promising that clinical trials focusing on manipulating autophagy to explore potential therapeutic strategies for improving adverse pregnancy outcomes will be developed in the near future.

## Conclusion and further perspective

Autophagy plays a critical role in the development of various diseases, including neurodegenerative diseases, autoimmune diseases, and cancer. Numerous studies have effectively elucidated the mechanisms linking autophagy to these conditions. However, consensus on the relationship between autophagy and pregnancy complications is lacking. Pregnancy complications are numerous, and our research focuses on major issues such as PE, GDM, RSA, and FGR. Other conditions, such as acute fatty liver of pregnancy and obstetric antiphospholipid syndrome, which can have significant adverse effects on both mothers and babies, are occasionally overlooked.

It is crucial to conduct more studies to establish a unified understanding of the link between autophagy and each pregnancy complication. Researchers should broaden their investigations to encompass a wider range of gestational disorders. This holistic approach will help develop a more comprehensive and nuanced understanding of the complex interplay between autophagy and challenges during pregnancy.
